# Reduction of NIFTI files storage and compression to facilitate telemedicine services based on quantization hiding of downsampling approach

**DOI:** 10.1038/s41598-024-54820-4

**Published:** 2024-03-02

**Authors:** Ahmed Elhadad, Mona Jamjoom, Hussein Abulkasim

**Affiliations:** 1 Department of Computer Science, Faculty of Computers and Information, South Valley University, Qena, Egypt; 2 Department of Computer Sciences, College of Computer and Information Sciences, Princess Nourah Bint Abdulrahman University, Riyadh, Saudi Arabia; 3 Department of Mathematics and Computer Science, Faculty of Science, New Valley University, El-Kharja, Egypt; 4 College of Engineering and Technology, University of Science and Technology of Fujairah, Fujairah, United Arab Emirates

**Keywords:** Medical image, NIFTI file, Compression, Downsampling, Upsampling, Data processing, Image processing, Communication and replication

## Abstract

Magnetic resonance imaging is a medical imaging technique to create comprehensive images of the tissues and organs in the body. This study presents an advanced approach for storing and compressing neuroimaging informatics technology initiative files, a standard format in magnetic resonance imaging. It is designed to enhance telemedicine services by facilitating efficient and high-quality communication between healthcare practitioners and patients. The proposed downsampling approach begins by opening the neuroimaging informatics technology initiative file as volumetric data and then planning it into several slice images. Then, the quantization hiding technique will be applied to each of the two consecutive slice images to generate the stego slice with the same size. This involves the following major steps: normalization, microblock generation, and discrete cosine transformation. Finally, it assembles the resultant stego slice images to produce the final neuroimaging informatics technology initiative file as volumetric data. The upsampling process, designed to be completely blind, reverses the downsampling steps to reconstruct the subsequent image slice accurately. The efficacy of the proposed method was evaluated using a magnetic resonance imaging dataset, focusing on peak signal-to-noise ratio, signal-to-noise ratio, structural similarity index, and Entropy as key performance metrics. The results demonstrate that the proposed approach not only significantly reduces file sizes but also maintains high image quality.

## Introduction

In recent years, the utilization of medical imaging has become an essential tool in clinical practices. Medical imaging is a technique and process utilized to observe the internal organs of the human body for diagnostic and treatment processes^1,2^. Medical imaging is used to examine and decline in mortality, fewer hospital admissions, longer life expectancy, shorter hospital stays, and reduced need for exploratory surgery. Different types of medical imaging are used to scan the human body, such as Magnetic resonance imaging, X-ray, Ultrasound imaging, Computed tomography Scanning, etc. Nowadays, the development and need for medical imaging modality have tremendously increased, and the need for producing, transferring, and sharing medical images has also been amplified^3,4^.

Telemedicine has emerged as a pivotal component of modern healthcare, offering a means to overcome geographical barriers, improve access to medical services, and facilitate timely medical interventions^5^. Central to the efficacy of telemedicine, particularly in diagnostics and treatment planning, is the reliance on medical imaging technologies. Magnetic Resonance Imaging (MRI), with its capability to produce high-resolution images critical for accurate medical assessments, plays a significant role in this domain^6^. However, this advancement comes with its own set of challenges, primarily associated with the management of MRI data, which is often stored in the Neuroimaging Informatics Technology Initiative (NIfTI) format. These files, characterized by their substantial size, pose significant challenges in terms of storage and transmission, especially in telemedicine scenarios where bandwidth and storage resources may be limited.

The need for efficient and effective telemedicine services is not just a matter of convenience but a crucial element in ensuring equal access to healthcare services, particularly in remote or resource-limited settings. The efficient transmission of large MRI files while maintaining the integrity and quality of the images is vital for accurate diagnosis and treatment planning. Current approaches to managing these large files, such as standard compression techniques, often lead to a trade-off between file size reduction and image quality. This compromise can impede the clinical utility of the transmitted images, potentially affecting patient outcomes.

Since medical images are stored in different formats by different modalities, retrieval, processing, and transmission are challenging tasks. The authors in^7^ described four main file formats widely used to store medical scans. They are NIfTI, Analyze, Medical Imaging NetCDF (MINC), and Digital Imaging and Communications in Medicine (DICOM). Medical Image file formats are categorized into two types. First is the format that wishes to standardize images generated by medical imaging modalities like DICOM. Second is different formats that aim to improve and expedite the post-processing analysis, like Analyze, MINC, and NIfTI.

It leads to insufficient bandwidth of the network and storage of memory devices. Medical images contain more details or information than simple text or document files, so they require extra demand of bandwidth to travel through different types of networks. Therefore, we have to minimize the volume of the images before the storing or transmitting processes. Here comes the need for compression. Compression is defined as minimizing the size/volume of data needed to describe a given amount of information. Therefore, efficient compression techniques are essential in the field of telemedicine and its applications^8,9^.

NIfTI is a file format developed in the 2000s to store neuroimaging, which retains the characteristics of the Analyze format while addressing its flaws^10^. Such a format employs some fallowed/underutilized fields in the Analyze 7.5 header for new data recording, such as picture orientation, to eliminate left–right ambiguity in brain studies. Moreover, NIfTI can also maintain different data types other than the Analyze format, such as unsigned 16-bit.

Despite the different files, header, and data pixels generated by such format, the outcome is one image file that is stored with the '.nii' extension. The files are 348 bytes for the header and the data pixels with the extensions'.hdr' and '.img,' and 352 bytes for one '.nii' file owing to the existence of four extra bytes at the end, primarily to make the size a divisible by 16, and guarantee a mechanism to for more metadata storing, where these four bytes are nonzero. In practice, an enhanced NIfTI format for diffusion-weighted magnetic resonance data processing has been developed.

The motivation behind this research stems from the growing need for efficient and effective telemedicine services, particularly in the realm of medical imaging. MRI plays a pivotal role in diagnostics and treatment planning, generating high-resolution images that are crucial for accurate medical assessments. However, the substantial size of MRI files, especially those in the NIfTI format, poses significant challenges in terms of storage and transmission, particularly in telemedicine scenarios where bandwidth may be limited. This issue is accentuated in remote or resource-limited settings, where access to advanced medical imaging and rapid communication between healthcare providers and patients is crucial.

Recognizing these challenges, our research is driven by the objective of developing a method that not only reduces the file size of MRI images without compromising their quality but also facilitates their swift and reliable transmission in telemedicine applications. By introducing an innovative downsampling approach using a quantization hiding technique coupled with a blind upsampling process for accurate reconstruction, we aim to address the pressing need for efficient and high-quality medical image communication. This advancement holds the potential to revolutionize the delivery of healthcare services, making high-quality medical imaging more accessible and improving the overall efficacy of telemedicine practices.

This study makes significant contributions to the field of medical imaging and telemedicine by:

- Introducing an innovative compression technique for NIfTI files using a quantization hiding of downsampling approach.
- Addresseing a critical gap in telemedicine, offering a solution that efficiently reduces file sizes without compromising the quality of MRI images.
- Enhancing telemedicine efficacy, particularly in bandwidth and storage-limited settings, ensuring high-quality image transmission vital for accurate diagnostics and patient care.
- Preserving the integrity of critical medical data and suggesting a comprehensive impact on digital healthcare solutions.

Through these contributions, our study presents a pivotal development in managing medical imaging data, facilitating improved healthcare delivery and access globally.

This paper proposes a new methodology for reducing the NIfTI file storage and compression to facilitate communication among health practitioners themselves. The proposed methodology uses the features of the discrete cosine transform (DCT) to embed two consecutive slice images to generate the stego slice. The performance of the proposed method was measured based on the MRI dataset, as well as the peak signal-to-noise ratio (PSNR), signal-to-noise ratio (SNR), structural similarity index (SSIM), and Entropy metrics. The paper is organized as follows: Section "Related work" surveys the work related to data-compressed techniques using the NIfTI format. Section "Materials and methods" discusses the materials and methods used. The proposed downsampling and upsampling approaches are described in Sect. "The proposed downsampling and upsampling approach". Section "Experimental results and discussion" presents the experiment results, the analysis of the results, and a comparison of the performance of the original and the retrieved NIfTI files. Finally, Section "Conclusion" summarizes the findings and conclusions.

## Related work

In the field of medical imaging and telemedicine, significant strides have been made to address the challenges of data management and transmission. Early studies primarily focused on conventional compression techniques to reduce the file size of medical images, yet often at the cost of image quality, which is crucial for diagnosis and treatment planning. For instance, Huffman encoding, a lossless compression algorithm, has been applied to medical images, offering some efficiency in storage without quality loss but with limitations in compression ratio^11^. Compression techniques have been a primary focus of research to tackle this issue. Huffman encoding, a lossless compression method, has shown effectiveness in medical image compression^12^. However, its efficiency varies with image size and complexity, especially for larger 3D MRI images. Block processing methods have also been explored^13^, but they often compromise critical image details, which is not viable for medical diagnostics. A novel index structure was presented to achieve speed up for compressing the data. The large files of neuroimaging data are compressed using the NIFTI format. The gzip-compression technique, which reduces the size of the data, is available. The compressed format can be accessed with high speed for performing the simple task of presenting the data and scrolling among time points. Depending on the user-configurable arrangement, the speed-up might vary between hundreds to thousands of times. The test findings show that this unique method has real-world applications in neuroimaging research. The neuroimaging data program is an enhanced library (libznz) that can read NIfTI files with additional indexes. The performance is demonstrated, which helps to avoid interference change^14^.

The study in^10^ has explored different medical imaging file formats like NIfTI, Analyze, MINC, and DICOM, each with distinct characteristics. For instance, the NIfTI format, an improvement over the Analyze format, is widely used for its ability to store neuroimaging data with enhanced data recording capabilities, including picture orientation and support for multiple data types. While these formats have facilitated the storage and analysis of medical images, the challenge of efficiently transmitting large file sizes over limited bandwidth networks, as often encountered in telemedicine scenarios, remains a concern. Meanwhile, in^15^, the authors illustrate the main types of medical image formatting. The first type is the desired format for standardizing pictures produced by the modalities of images such as DICOM. Second, formats like Analyze, MINC, and NIfTI are designed to make post-processing analysis easier and more powerful. Huffman encoding is a lossless compression algorithm, and the occurrence of the symbol is based on the frequency. The performance of metrics, such as compression ratio, percentage, and bit per pixel, is evaluated using the Huffman algorithm. It can be modified to get high compression output for 3D images. The most common modeling methodology and attack tree were used to build a risk assessment method with the attack occurrence probability (AOP) and assault success probability (ASP) as variables and evaluated the benefits and possible limits of the method.

Recent developments have seen the incorporation of AI and machine learning into image processing. The application of Variational Autoencoders (VAEs) for tumor identification in 2-D MRI images represents a notable advancement^16^. These self-learning models harness the power of artificial intelligence to not only compress data but also enhance feature extraction and image analysis capabilities. Such advancements indicate a shift towards integrating AI and machine learning techniques for more efficient image processing, compression, and diagnostic accuracy. The NIfTI file format, widely adopted for neuroimaging, emerged as a solution to limitations in earlier formats like Analyze. It offers enhanced capabilities for data recording and supports multiple data types^17^. Despite these advancements, efficient transmission of these large files remains a challenge, particularly in telemedicine contexts where bandwidth is limited^18^.

A heterogeneous platform is developed for analyzing and storing MRI data, which is created to process automatically with the subsequent visualization^19^. In^20^, the module will be utilized to organize classes for training on neuroimaging. The proposed model is used to analyze the MRI data of the laboratory animal. Data processed and analyzed will be stored in the NIfTI file and then separated into a text file. The file in NIfTI is used for processing and analyzing. The proposed module is utilized for the neuroimage training process. However, the challenge of efficiently transmitting large neuroimaging files, particularly in NIfTI format, remains a largely unresolved issue. While studies have explored various compression algorithms, there's a gap in research that specifically targets the compression of NIfTI files for telemedicine use without compromising image quality. The existing literature often overlooks the unique requirements of telemedicine, such as the need for efficient data transmission over limited bandwidth and the necessity to maintain high image fidelity for accurate remote diagnosis. Current methods still grapple with either data loss in lossy compression or insufficient size reduction in lossless methods, limiting their practicality in real-time telemedicine^13^.

The basic concept of an image file is examined with some format that mainly describes the usage of medical images, pixel data, and interpretation of data. The pixel number is utilized to examine the field view of the acquisition modality. The photometric interpretation specified the interpreted data for displaying the correct image. The medical image file format is used to standardize the image generated by the modalities for image diagnosis^21^. NIfTI format is used to store the image orientation in the space of the image volume. The default file format is doing the analysis, and the NIfTI file format is used for storing the analyzed neuroimage. The modalities are diagnosed by encoding the image file. A compression-decompression technique is proposed in which each image is segmented into non-overlapping blocks to achieve the benefits of block processing. The most frequent pixel is predicted, and occurrences are deleted permanently for 4 × 4 blocks. The decoding stage has completely regenerated the block in which others are encoded.

Our research addresses this gap by introducing a novel quantization hiding downsampling technique specifically designed for NIfTI files. This method achieves significant file size reduction and maintains the high quality and integrity of MRI images, which is crucial for accurate medical diagnosis. While previous research in medical imaging has laid a strong foundation for data management and analysis, our study builds upon this by addressing the specific challenges associated with the compression and transmission of NIfTI files in telemedicine.

## Materials and methods

### A. OpenNeuro dataset ds003799

In this paper, we propose a down-sampling method to reduce the multi-dimensional neuroimaging data size using the concept of data hiding by quantization hiding technique. The OpenNeuro ds003799 dataset is used in this work. Such a data set is used in a study to test the effect of running sports on decreasing and minimizing the symptoms of depression and increasing the Hippocampal volume for young adults over 14 days^22,23^. The study was approved by the University of Graz's authorized ethics committee. Informed consent was obtained from all individual participants involved in the study. The data supporting this dataset findings are available online under OpenNeuro Dataset ds003799. This study's running intervention was divided into seven units, each lasting 50–60 min, and carried out over two weeks. The typical running path was about five kilometers long and led through a largely woodland region at a local leisure park. Two groups of volunteers shared in this experiment were examined at three different periods. In this dataset, a total of 68 people were enlisted. Out of this group, 48 people completed all of the requisite MRI scans and psychometric tests and took part in the running intervention. Participants said they exercise for approximately 30 min weekly (M = 0.53; SD = 1.2). They were randomly allocated to one of two intervention groups, each receiving a time-delayed intervention. The first group (the intervention group) ran between the first (t1) and second (t2) test sessions, whereas the second group (the waiting group) got training between the second and third test sessions (t3). The German version of the Center for Epidemiological Studies Depression Scale was used at each time point of evaluation (t1, t2, and t3). In^24^, it was administered to test intervention-related changes in depressive symptoms. The dataset includes the MRI data of n = 48 participants who completed all three MRI scans (at t1, t2, t3). Specifically, for every single participant (e.g., sub-season101), three subfolders of MRI data ('ses-1', 'ses-2', and 'ses-3' for each time point of assessment) are available.

### B. NII (or NIfTI) files

As of this writing, NII (or NIfTI) files are the most used format for multi-dimensional neuroimaging data^25^. NIfTI is a raster format that requires at least 3-dimensional data in the form of voxels, or pixels having a width, height, and depth. The first four dimensions are defined as three spatial dimensions and time, with the other dimensions being utilized for other things, but the time dimension is frequently used to convey anything other than time^26^.

The NIfTI file format stores the orientation of image volume space and allows it in a double way. The translation is used to map the coordinates of the voxel to frame the reference and define the image's alignment with the standard system. The NIfTI format adopted the default format of widely used public domain software packages such as SPM, FSL, and AFNI. The extension NIfTI file format is used for processing the diffusion-weighted magnetic resonance data^27^.

## The proposed downsampling and upsampling approach

**Figure 1:**
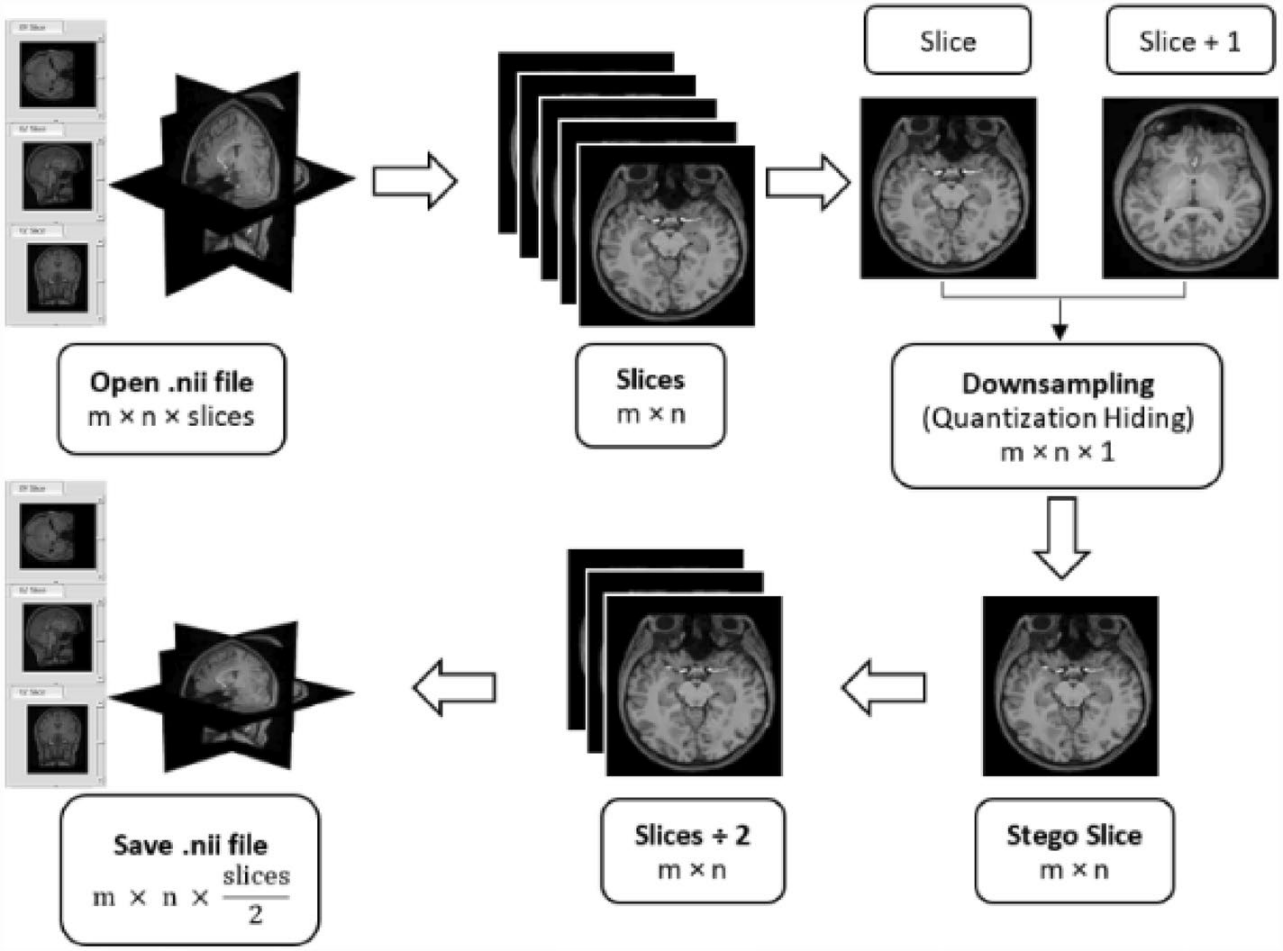
The proposed downsampling overview (Quantization Hiding).

Figure 1 shows a high-level view of the main part, which uses the quantization hiding technique of the proposed downsampling technique to embed a slice image into another slice image of NIfTI files. The primary idea behind this approach is to create a new slice picture (stego slice) with pixels that are a composite of two identical slice images; the cover and the other are used as the hidden message (Msg). Similar to the cove image (the first slice), the resulting stego slice picture should have as low distortion as possible. Then, without the availability of these slice images, this stego slice picture might be utilized blindly to construct the embedded next slice image. The proposed downsampling method first opens the NIfTI file as volumetric data with the dimension $m\times n\times slices$. Then, it plans the volumetric data into the number of slice images of the size $m\times n$. Subsequently, the quantization hiding technique will be applied on each two consecutive slice images to generate the stego slice with the same size. Finally, it assembles the resultant stego slice images to produce the final NIfTI file as volumetric data with the dimension $m\times n\times \frac{slices}{2}$.

**Figure 2:**
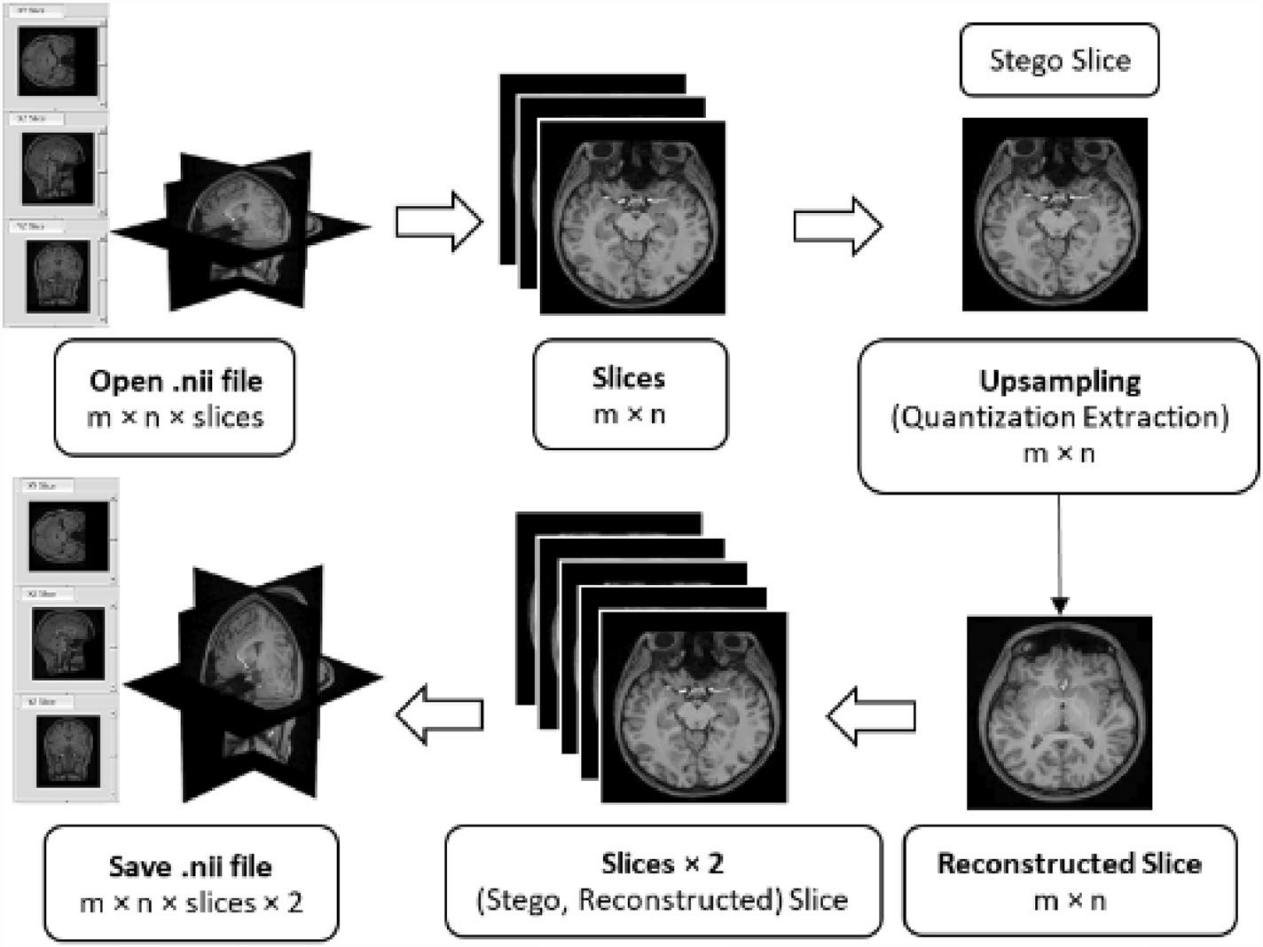
The proposed upsampling overview (Quantization Extraction).

In the upsampling process, the steps of the downsampling process are generally reversed to recover the next slice image, as depicted in Fig. 2. The process starts by opening the stego NIfTI file as volumetric data and then preparing the volumetric data into the number of slices images. So, the reconstructed slice can be extracted using the quantization extraction technique. Finally, the final reconstructed NIfTI file will be assembled from the Stego and reconstructed slice images, respectively.

### The quantization hiding and extraction technique

Initially, the cover and Msg pictures will be the current and next slice images, respectively. The Cover and Msg slice pictures are normalized in the preprocessing step to provide float pixel values with float values in the range [0, 1] rather than the integer range. Consequently, normalization changes the slice image $Slice : \left\{{X\subseteq {\mathbb{R}}}^{d}\right\}\to \left\{Min,\cdots , Max\right\}$ from the range values $\left[Min, Max\right]$ into a new slice image $Normalized(Slice) : \left\{{X\subseteq {\mathbb{R}}}^{d}\right\}\to \left\{0,\cdots , 1\right\}$. The following Eq. 1 represents the linear normalization of the slice image:

1 $$\begin{array}{c}Normalization\left(Slice\right)= \frac{Slice-Min}{Max-Min}\end{array}$$

The resultant normalization ($Slice$) and normalizing ($Slice+1$) pictures are separated into several sections of $4\times 4 matrices$, which are called microblocks after normalization (MB). If the dimensions of the image are not divisible by four, the technique ignores the last MB of the image without making any adjustments.

The main goal of this phase in the quantization concealment process is to incorporate the normalized slice + 1 MB (Msg) into the matching normalized slice MB (Cover) to create a combined stego slice MB picture (stego). To begin, the cover's MBs are subjected to a 2D DCT as follows in Eq. 2:

2 $$\begin{array}{c}DCT coefficients=T \times M{B}_{Cover}\times {T}^{\mathrm{^{\prime}}}\end{array}$$

$$T= \left[\begin{array}{cccc}\frac{1}{\sqrt{4}}& \frac{1}{\sqrt{4}}& \frac{1}{\sqrt{4}}& \frac{1}{\sqrt{4}}\\ \sqrt{\frac{2}{4}} Cos(\frac{\pi }{8})& \sqrt{\frac{2}{4}} Cos(\frac{3\pi }{8})& \sqrt{\frac{2}{4}} Cos(\frac{3\pi }{8})& \sqrt{\frac{2}{4}} Cos(\frac{3\pi }{8})\\ \sqrt{\frac{2}{4}} Cos(\frac{3\pi }{8})& \sqrt{\frac{2}{4}} Cos(\frac{3\pi }{8})& \sqrt{\frac{2}{4}} Cos(\frac{3\pi }{8})& \sqrt{\frac{2}{4}} Cos(\frac{3\pi }{8})\\ \sqrt{\frac{2}{4}} Cos(\frac{3\pi }{8})& \sqrt{\frac{2}{4}} Cos(\frac{3\pi }{8})& \sqrt{\frac{2}{4}} Cos(\frac{3\pi }{8})& \sqrt{\frac{2}{4}} Cos(\frac{3\pi }{8})\end{array}\right].$$

$T$ Represents the DCT transform matrix, and its transpose is denoted by ${T}{\prime}$. The DCT coefficient is the result obtained in the dot product utilized in the matrix product. There are 4 × 4 coefficients in the final matrix: one calculation and fifteen detailed coefficients. Following that, a substitution process is made to embed the Cover DCT coefficients with the relevant fuzzified Msg's MB pixel values using the following Eq. 3:

$$Stego\, coefficient= sign\left(DCT\, coefficien{t}_{Cover}\right)\left[\frac{2}{\beta }\left(M{B}_{Msg}+i\right), \frac{2i}{\beta }\le \left|DCT\, coefficien{t}_{Cover}\right|\le \frac{2\left(i+1\right)}{\beta }\right]$$

3 $$\begin{array}{c}\forall i=\mathrm{0,1},2, \dots , \beta -1\end{array}$$

where β fulfills the cover coefficients on the interval [0, 4], which is the total number of intervals, the ${\text{sign}}$ function returns the value 1 or -1 when the relevant component is greater than or less than 0. The DCT coefficients' absolute values fall within the range [0, 4], with the range set by the lowest and highest for each coefficient value. As a result, it will be employed as an extra parameter to split the absolute value range [0, 4] of the cover DCT coefficients. During the embedding process, the estimated coefficient value of the cover MB is maintained constant for each MB to enhance stego slice picture quality against noise. The Stego slice image is attained after the embedding process by using the inverse DCT using the following Eq. 4:

4 $$\begin{array}{c}M{B}_{Stego}={T}^{\mathrm{^{\prime}}}\times DC{T}_{Stego}\, coefficients \times T\end{array}$$

Finally, the Stego slice picture is denormalized to restore the original domain of the pixel values. On the other hand, the stages of the quantization extraction procedure are exactly the opposite of those taken during the quantization concealment phases. As a result, these operations begin by using normalization to transform pixel data to the [0, 1] domain. In the second stage, the normalized Stego slice image is partitioned into 4 × 4 MB, and the following Eq. 5 is used to compute the DCT decomposition:

5 $$\begin{array}{c}DCT\, coefficients=T \times M{B}_{Stego}\times {T}^{\mathrm{^{\prime}}}\end{array}$$

The Stego DCT coefficient is the extraction stage according to the third step, as shown as follows in Eq. 6:

$$M{B}_{RecMsg}=\frac{\beta }{2}\left(DCT\, coefficien{t}_{Stego}- \frac{2i}{\beta }\right), \frac{2i}{\beta }\le \left|DCT\, coefficien{t}_{Stego}\right|\le \frac{2\left(i+1\right)}{\beta },$$

6 $$\begin{array}{c}\forall i=\mathrm{0,1},2, \dots ,\beta -1\end{array}$$

where β is the same integer number employed during quantization, additionally, the mean value of two neighboring elements is computed and substituted on the first element in $M{B}_{RecMsg}$ to rebuild it appropriately. The pixel values are then denormalized to return to their original domain in the resulting rebuilt slice. Finally, to enhance the visual quality of the reconstructed slice image, a 3-by-3 neighborhood median filter is applied to each pixel of the slice image surrounding the consistent pixel.

We developed two distinct algorithms to compress and decompress MRI data stored in NIfTI files efficiently, which is crucial for enhancing telemedicine services. As illustrated in Algorithm 1, The Downsampling Algorithm focuses on compressing the MRI data. This process begins by normalizing each slice of the NIfTI file to a standard range and then dividing them into 4 × 4 micro-blocks. These blocks undergo a 2D Discrete Cosine Transform (DCT) for frequency domain conversion. The core of this algorithm is the quantization hiding technique, where consecutive slices are merged, embedding one slice into another to create a 'stego' slice with minimal distortion. After applying the inverse DCT, these stego slices are assembled to form the compressed NIfTI file. Conversely, in Algorithm 2, the Upsampling Algorithm serves to decompress the data. It starts by processing each stego slice from the compressed file, extracting the embedded slice using a quantization extraction technique. Following inverse normalization, the extracted slices are reassembled, reconstructing the original MRI data. These algorithms, when combined, provide a robust solution for handling large neuroimaging files, significantly reducing their size while preserving essential diagnostic details, thereby facilitating more efficient and effective telemedicine services.

**Algorithm 1:**
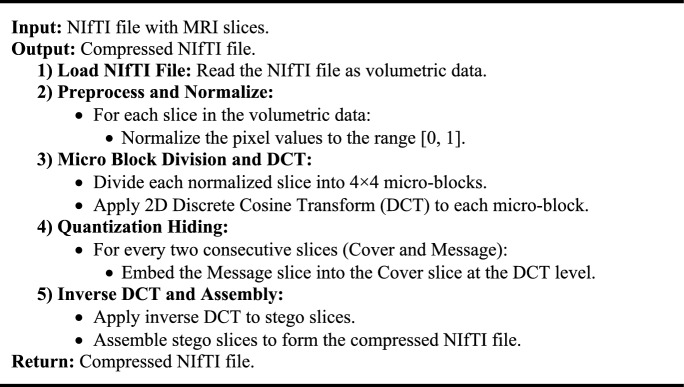
Downsampling for NIfTI File Compression.

**Algorithm 2:**
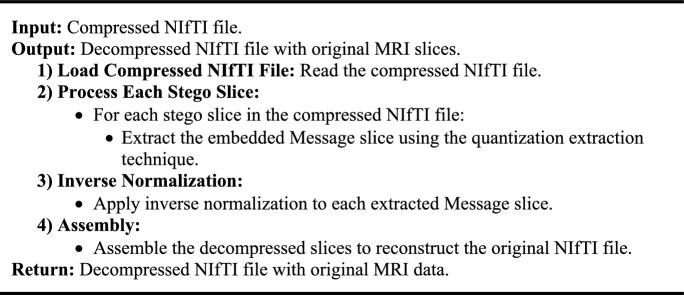
Upsampling for NIfTI File Decompression.

## Experimental results and discussion

The description metrics were utilized to implement and assess the suggested technique using the OpenNeuro ds003799 dataset (version: 2.0.0) in this section. This dataset was mentioned and downloaded from^23^, and it contains 48 individuals' data and 144 NIfTI files in total, for every single participant 3 subfolders, 224 × 256 × 256 the image size of each MRI and 2.03 GB total files size. For the DCT process in the quantization hiding technique, a kernel size of 4 × 4 has been optimized. The stride configuration is set to a stride of 1, which is essential for ensuring comprehensive pixel processing and maintaining the integrity of the image. Regarding loss functions, PSNR, SNR, and Bit error rate (BER) are used for fidelity assessment, while SSIM and Entropy are employed to evaluate the perceptual quality of the images. The MATLAB version: 9.9.0.1467703 (R2020b) is used for implementing the simulation with the toolbox of image processing and comprises reading the metadata and volumetric data facilities for NIfTI file importing and processing.

**Figure 3:**
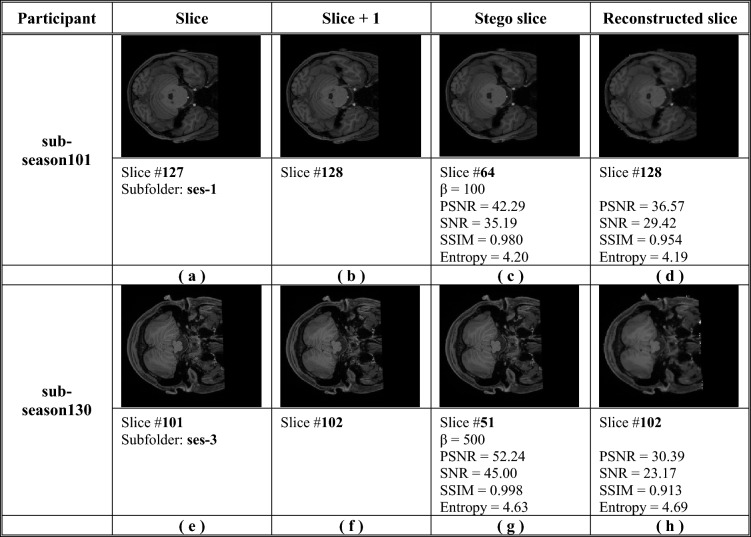
Slice images samples of participants' sub-season101' and 'sub-season130' and the results Stego slice images of the quantization hiding method.

Figure 3 illustrates the original two-slice images, stego slice image, and reconstructed slice image samples from two participants: 'sub-season101' and 'sub-season130' slices of the proposed method. Figure 3a, b, e, and f shows the original slice pictures for participants' sub-season101' and 'sub-season130,' respectively. Figure 3c and g, however, show the resultant slice after quantization, which hides the next slice inside the present slice. Figure 3 and h illustrate the rebuilt slice after an authorized person used the quantization extraction technique to a Stego slice. A simple visual evaluation of the findings reveals that the Stego slice photos are of excellent quality, while the recovered slice images are of acceptable quality.

To assess the performance of the proposed algorithm for each slice, we employed the PSNR, SNR, and SSIM^28,29^. The PSNR and SNR are methods for determining the quality of invisibility observation. On the other hand, the SSIM index is a perceptual metric that assesses picture quality decline as perceived structural information changes^30,31^. These metrics provide insights into the quality of invisibility observation and perceptual changes in the structural information of the images.

The PSNR, a widely used metric in image compression, is defined as Eq. 7:

7 $$\begin{array}{c}PSNR=10{{\text{log}}}_{10}\left(\frac{{peakval}^{2}}{\frac{1}{MN}\sum_{i=0}^{M-1}\sum_{j=0}^{N-1}{\left(f\left({x}_{i},{y}_{j}\right)-g\left({x}_{i}, {y}_{j}\right)\right)}^{2}}\right)\end{array}$$

where f(x, y) is the NIfTI file's original slice and g(x, y) is the stego slice pictures; the height and breadth of the slice pictures are M and N. The $peakval$ is the picture datatype's range (e.g., the value is 65,535 for the uint16 image). Higher PSNR values generally indicate better-quality invisibility. In addition to PSNR, SNR is another crucial metric that quantifies the level of the desired signal relative to the level of background noise^32^. It is calculated as Eq. 8:

8 $$\begin{array}{c}SNR=10{{\text{log}}}_{10}\left(\frac{\sum_{i=0}^{M-1}\sum_{j=0}^{N-1}{f\left({x}_{i},{y}_{j}\right)}^{2}}{\sum_{i=0}^{M-1}\sum_{j=0}^{N-1}{\left(f\left({x}_{i},{y}_{j}\right)-g\left({x}_{i}, {y}_{j}\right)\right)}^{2}}\right)\end{array}$$

The SSIM index, on the other hand, is a perceptual metric that evaluates image quality degradation as perceived structural information changes. It is given by the formula in Eq. 9:

9 $$\begin{array}{c}SSIM\left(x,y\right)= \frac{\left(2{\mu }_{x}{\mu }_{y}+{c}_{1}\right)\left(2{\sigma }_{xy}+{c}_{2}\right)}{\left({\mu }_{x}^{2}+{\mu }_{y}^{2}+{c}_{1}\right)\left({\sigma }_{x}^{2}+{\sigma }_{y}^{2}+{c}_{2}\right)}\end{array}$$

where µx and µy are the local means of x and y, ${\sigma }_{x}^{2}$ and ${\sigma }_{y}^{2}$ are the variances, ${and \sigma }_{xy}$ is the cross-covariance. The two variables, c1, and c2, are variables to stabilize the division with a weak denominator.

**Figure 4:**
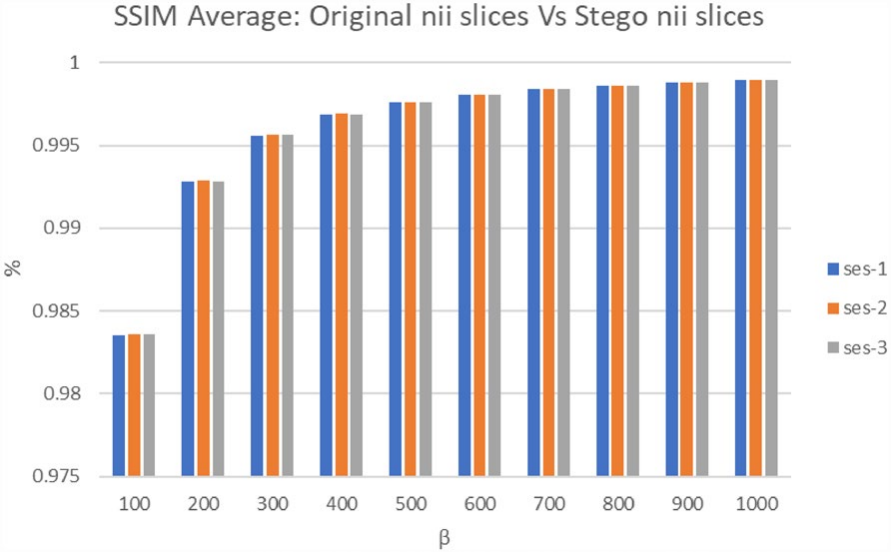
The average of SSIM for the original slice and Stego slice images.

Tables 1 and 2 examine the performance comparison of the original slice and the stego slice images in decibels (dB) to the resulting average PSNR and average SNR values for each of the three subfolders of participants' MRI data ("ses-1", "ses-2", and "ses-3"). The average PSNR values are 53.59 dB, 53.60 dB, and 53.55 dB of "ses-1", "ses-2", and "ses-3" respectively for β = 500. PSNR has a minimum of 43.80 dB ("ses-3" and a value of 100) and a maximum of 58.11 dB ('ses-2' and a value of 1000). In general, greater PSNR values denote the invisibility of higher quality. Similarly, the average SNR values for β = 500 are 46.46 dB, 46.46 dB, and 46.41 dB for the respective three subfolders of participants' MRI data, reaching up to 50.96 dB for β = 1000. These values indicate that higher SNR values correspond to higher quality invisibility and clearer signals in the stego images. Figure 4 displays the average SSIM values obtained for 48 individuals for various values between the original and stego slice pictures. For = 500, the average SSIM values were between 0.99760 and 0.99762. While the lowest and maximum values of SSIM for = 100 and = 1000, respectively, demonstrate that the greatest value creates a high-quality stego image.

**Table 1:** The average of PSNR for the original slice and stego slice images.

β	ses-1	ses-2	ses-3
100	43.86	43.85	43.80
200	47.83	47.83	47.78
300	50.33	50.33	50.28
400	52.16	52.16	52.11
500	53.59	53.60	53.55
600	54.77	54.78	54.73
700	55.77	55.78	55.73
800	56.63	56.64	56.60
900	57.41	57.41	57.37
1000	58.10	58.11	58.06

**Table 2:** The average SNR for the original slice and stego slice images.

β	ses-1	ses-2	ses-3
100	36.73	36.71	36.66
200	40.70	40.69	40.64
300	43.19	43.19	43.14
400	45.02	45.02	44.97
500	46.46	46.46	46.41
600	47.63	47.64	47.58
700	48.63	48.64	48.58
800	49.50	49.50	49.45
900	50.27	50.27	50.22
1000	50.96	50.96	50.92

**Figure 5:**
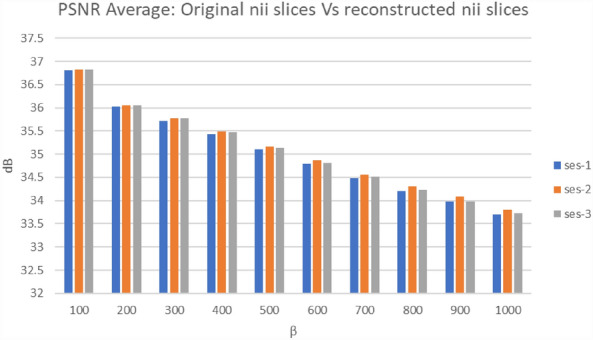
The average of PSNR for the original slice and reconstructed slice images.

**Figure 6:**
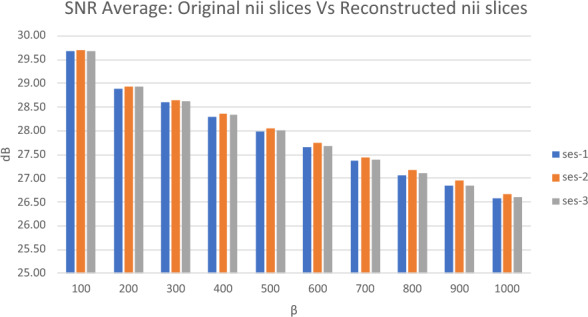
The average of SNR for the original slice and reconstructed slice images.

To evaluate the performance of the extraction process in the proposed algorithm, both PSNR, SNR, SSIM and Entropy measurements were utilized. These metrics provide insights into the quality of the reconstructed slice images compared to the original ones. As indicated in Fig. 5, the average PSNR values for the comparison between the original and reconstructed slice images varied from 33.70 to 36.82 dB across different β values. These readings suggest a certain level of quality in the reconstructed images, with higher PSNR values generally indicating better reconstruction fidelity. In addition to PSNR, SNR was also measured to evaluate the extraction performance. As shown in Fig. 6, the average SNR values for the comparison between the original and reconstructed slice images varied across different β values in the three subfolders of participants' MRI data (ses-1, ses-2, and ses-3). For β = 100, the SNR values were around 29.69 dB, decreasing gradually to 26.57 dB for β = 1000 in ses-1. This trend was consistent across all subfolders. Generally, a higher SNR indicates a clearer signal amidst noise, thus suggesting a relative decrease in signal clarity in the reconstructed images as β increases. Additionally, Table 3 presents the average SSIM values obtained to evaluate the reconstructed and original slice images. The SSIM values ranged from 0.9499 to 0.9585 on average, with the highest values observed at β = 100 (0.9585) and the lowest at β = 1000 (0.9499). The SSIM metric, being a measure of structural similarity, indicates how well the structural information of the original image is preserved in the reconstructed image. The high SSIM values suggest that the structural integrity of the images is largely maintained during the extraction process, although a slight decrease is noted with increasing β.

**Table 3:** The average of SSIM for the original slice and reconstructed slice images.

β	ses-1	ses-2	ses-3
100	0.9583	0.9585	0.9583
200	0.9569	0.9571	0.9570
300	0.9568	0.9571	0.9569
400	0.9563	0.9566	0.9563
500	0.9554	0.9557	0.9554
600	0.9543	0.9547	0.9543
700	0.9532	0.9536	0.9532
800	0.9521	0.9526	0.9521
900	0.9511	0.9516	0.9510
1000	0.9499	0.9504	0.9499

In the context of image processing, Entropy is a fundamental concept derived from information theory that quantifies the amount of information or randomness contained in an image^33^. Essentially, it measures the unpredictability or uncertainty of the image data. The Entropy of an image is calculated based on the probability distribution of the intensity levels (or pixel values) within the image. Mathematically, for a grayscale image, entropy H is defined as Eq. 10:

10 $$\begin{array}{c}H=-\sum_{i=0}^{L-1}P\left(i\right){{\text{log}}}_{2}P\left(i\right)\end{array}$$

where L is the number of possible intensity levels in the image, P(i) is the probability of occurrence of intensity level i, and the logarithm is to the base 2, reflecting the binary nature of digital information. The probability P(i) is typically computed as the frequency of the intensity level i divided by the total number of pixels in the image. In practical applications, Entropy is used as a tool to analyze the texture and content of an image. For instance, in medical imaging, Entropy can be employed to assess the quality and information content of an image, which is crucial for accurate diagnosis.

Table 4 presents the average entropy values for original, stego, and reconstructed slice images across different β values for each of the three subfolders of participants' MRI data. The Entropy, a measure of randomness or unpredictability in the image data, provides insight into the effect of the quantization hiding and extraction process on image information content. The entropy values for the original, stego, and reconstructed images are relatively consistent across all three subfolders of participants' MRI data. This consistency indicates that the quantization hiding technique and the reconstruction process maintain a stable impact on the information content of the images across different datasets or conditions. As β increases, there is a noticeable trend in the Entropy of the stego and reconstructed images. Initially, for lower values of β (100 to 600), the Entropy of the stego images decreases slightly compared to the original, while the reconstructed images show a marginal decrease or remain relatively stable. This suggests a minor loss of information or predictability due to the embedding process. However, for higher values of β (700–1000), the Entropy of both stego and reconstructed images begins to increase, eventually surpassing the Entropy of the original images. This trend could be indicative of the increasing complexity or randomness in the image data as the quantization parameter increases, possibly due to the embedding of more detailed information from the message image. The fact that the Entropy of the reconstructed images approaches or exceeds that of the original images at higher β values is particularly noteworthy. This could imply that the reconstruction process is effective in retaining or even enhancing the informational content of the images, which is a positive indicator of the quality of the reconstructed images.

**Table 4:** The average of Entropy for the original slice, stego slice, and reconstructed slice images.

*β*	ses-1			ses-2			ses-3		
	Original	Stego	Reconstructed	Original	Stego	Reconstructed	Original	Stego	Reconstructed
100	3.981	3.975	3.950	3.976	3.968	3.943	3.984	3.975	3.950
200	3.981	3.966	3.953	3.976	3.959	3.946	3.984	3.966	3.954
300	3.981	3.962	3.954	3.976	3.955	3.946	3.984	3.963	3.954
400	3.981	3.960	3.954	3.976	3.953	3.947	3.984	3.961	3.955
500	3.981	3.959	3.956	3.976	3.952	3.949	3.984	3.959	3.957
600	3.981	3.959	3.959	3.976	3.952	3.951	3.984	3.959	3.959
700	3.981	3.960	3.965	3.976	3.953	3.957	3.984	3.960	3.965
800	3.981	3.962	3.966	3.976	3.955	3.958	3.984	3.962	3.966
900	3.981	3.964	3.970	3.976	3.957	3.962	3.984	3.964	3.970
1000	3.981	3.965	3.970	3.976	3.958	3.962	3.984	3.965	3.970

**Figure 7:**
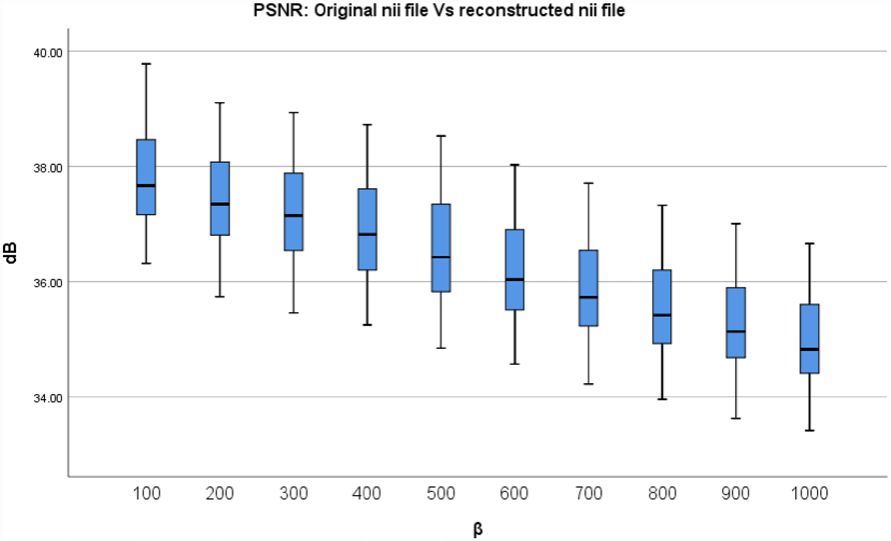
The PSNR values between the finished reconstructed files and the original NIfTI files are shown in a box plot.

**Figure 8:**
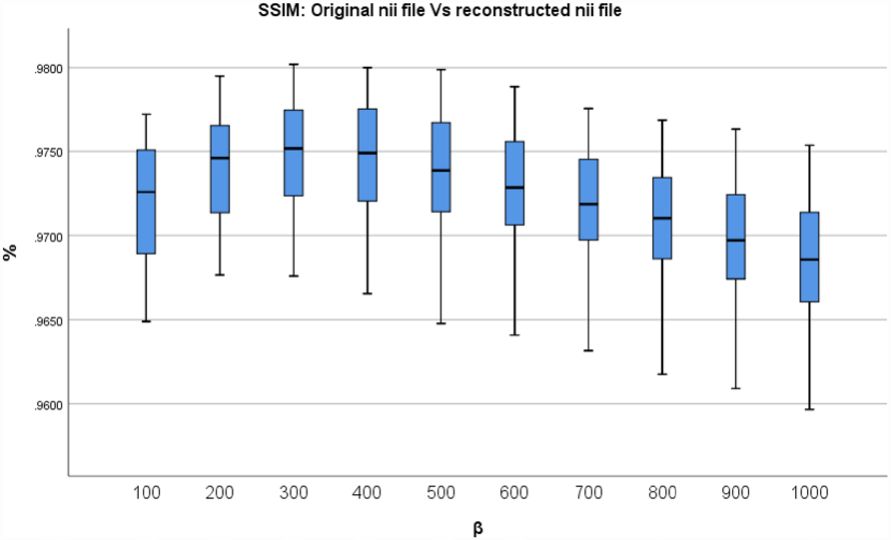
The Box plot of the SSIM values between the final reconstructed and the original NIfTI files.

In descriptive statistics, a box plot is a standard method for representing the distribution of data based on a five-number summary (minimum, first quartile, or 25th percentile (Q1), median, third quartile, or 75th percentile (Q3), and maximum). It is a graphical method for displaying numerical data's locality, dispersion, and skewness through their quartiles^34,35^. To conclude the evaluation between the final reconstructed and original NIfTI files for various β values, Figs. 7 and 8 depict the resultant box plot diagrams of the PSNR and SSIM. The PSNR value for β = 500, the minimum value is 34.85 dB, the median value is 36.58 dB, the maximum value is 38.53 dB, the 25th percentile value is 35.82 dB, and the 75th percentile value is 37.35 dB. While the SSIM value for β = 500, the minimum value is 0.9648, the median value is 0.9739, the maximum value is 0.9799, the 25th percentile value is 0.9714, and the 75th percentile value is 0.9767.

**Figure 9:**
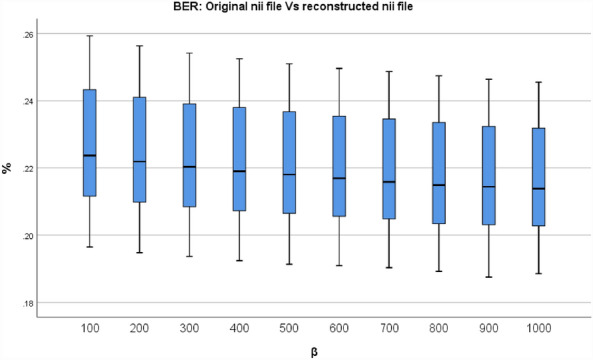
The Box plot of the BER values between the final reconstructed and the original NIfTI files.

Finally, a scalar with a Bit Error Rate (BER) is returned, which is the bit error of the total number of bits used in the binary form. Figure 9 shows the box plot of the BER evaluation between the final reconstructed and the original NIfTI files for various β values. The BER value for β = 500, the minimum value is 0.191%, the median value is 0.221%, and the maximum value is 0.251%, the 25th percentile value is 0.206%, the 75th percentile value is 0.237%.

**Figure 10:**
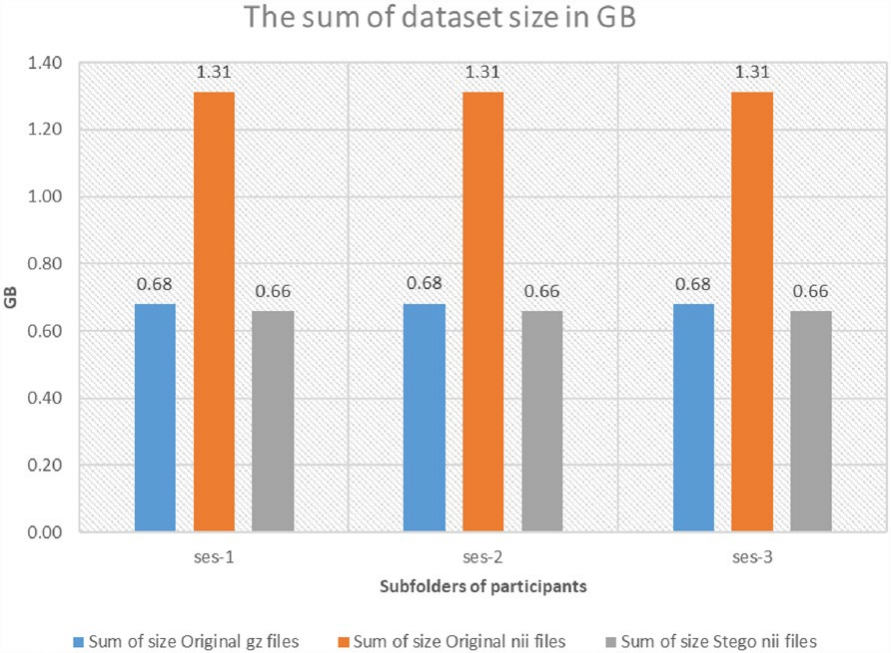
The total size sum of the subfolders of participants' MRI data files.

The most prevalent image compression techniques fall into two categories: Lossy compression and lossless compression. Moreover, the details of the image or quality of the image are lost in lossy compression, whereas in lossless compression, there is no information loss, and the image remains the same as the original, with less storage space. Lossless compression is often required when compressing medical images due to the high value of the information contained in the images, and it is important to preserve those details during compression. Therefore, this study reduces the NIfTI file storage and compression to facilitate communication among health practitioners themselves and with their patients in telemedicine services. Therefore, Fig. 10 shows the total size of the subfolders of participants' MRI data ('ses-1', 'ses-2', and 'ses-3') in Gigabytes (GB). The proposed technique provides a better compression ratio when compared to the original NIfTI and the GZ compressed files by the standard GNU zip. So, the proposed technique provides a better compression ratio when compared to other existing techniques.

Hence, we present a detailed analysis of the complexity of the proposed downsampling and upsampling approach. The complexity assessment encompasses both the downsampling and upsampling processes, considering key steps such as quantization hiding, extraction, and associated transformations. The downsampling process begins by opening the NIfTI file as volumetric data with dimensions $m\times n\times slices$. The data is then planned into a number of slice images of size $m\times n$. This initial step involves minimal computational complexity and is linear with respect to the size of the input volumetric data. The quantization hiding technique is applied to each pair of consecutive slice images. This involves the following major steps: Normalization and MB generation, DCT transformation, Quantization concealment, and final assembly.

Cover and Msg slice images undergo normalization, transforming pixel values into the range [0, 1]. The normalization process has a linear complexity with respect to the number of pixels in the slice images. The normalized images are divided into 4 × 4 microblocks (MB), and if the dimensions are not divisible by four, the last MB is ignored. The generation of microblocks has a linear complexity. The DCT transformation is applied to Cover MBs, introducing a complexity proportional to the number of pixels in each MB, particularly the DCT, which has a complexity of O(N log N) for each 4 × 4 MB within the slice images. The embedding process involves quantization concealment, which includes computing Stego coefficients, applying inverse DCT, and denormalizing the resulting slice. The complexity of these operations is linear with respect to the size of the microblocks. The resultant stego slice images are assembled to produce the final NIfTI file with dimensions $m\times n\times \frac{slices}{2}$. The assembly has a linear complexity.

In the same context, the complexity of the upsampling approach is primarily governed by the extraction process, which mirrors the downsampling steps but in reverse. The upsampling process begins by opening the stego NIfTI file as volumetric data and preparing it into the number of slice images. The quantization extraction technique involves the following major steps: Normalization, DCT transformation, Quantization extraction, and Final reconstruction. The complexity of these operations is linear with respect to the size of the microblocks. The overall complexity of the proposed downsampling and upsampling approach is dominated by the computational cost of the DCT transformations, quantization concealment, and extraction operations. The linear complexity of these steps with respect to the size of the microblocks and the number of slices contributes to an overall linear complexity for the entire process. The proposed approach demonstrates computational efficiency, making it suitable for practical implementation in telemedicine applications.

We conducted a comparative analysis with current state-of-the-art compression methods to highlight the efficacy of our proposed quantization hiding of downsampling technique for NIfTI files. Recent advancements in this field, such as the utilization of advanced codecs like JPEG2000 or sophisticated AI-driven approaches like deep learning-based compression^36,37^, have set high benchmarks in balancing compression efficiency and image quality. However, our method surpasses these models by achieving higher compression ratios without compromising the diagnostic quality of MRI images. For instance, while JPEG2000 offers significant compression, it tends to introduce artifacts in higher compression settings^37^. Similarly, AI-based methods, though efficient, require substantial computational resources and training data. In contrast, our approach demonstrates superior performance with PSNR values up to 58.11 dB and SSIM values in the range of 0.9499–0.9585, indicating minimal loss of image fidelity. Additionally, the quantization hiding technique ensures a more straightforward implementation and lower computational overhead, making it more suitable for real-time telemedicine applications. This comparative analysis underscores the potential of our method as a more effective solution for medical image compression in the context of telemedicine.

## Conclusion

In this paper, we presented a novel approach for the reduction of NIfTI file storage and compression to facilitate telemedicine services, employing a quantization hiding of downsampling technique. Our method primarily focuses on embedding a slice image into another slice image of NIfTI files, thereby creating a new stego slice picture with minimal distortion compared to the original. This technique was designed to enhance the efficiency of telemedicine services by reducing the storage and bandwidth requirements for transmitting neuroimaging data. The downsampling process involved a quantization hiding technique that normalized the slice images and embedded them into each other using an algorithm based on Discrete Cosine Transform (DCT). The upsampling process, essentially the inverse of downsampling, utilized a quantization extraction technique to recover the next slice image from the stego slice.

The comprehensive evaluation of this approach using an MRI dataset and metrics such as Peak Signal to Noise Ratio (PSNR), Signal to Noise Ratio (SNR), and Structural Similarity Index (SSIM) has demonstrated its efficacy. Notably, the method achieved high PSNR and SNR values and maintained SSIM values within a range indicative of excellent image quality preservation. For instance, we observed PSNR values ranging up to 58.11 dB and SSIM values between 0.9499 and 0.9585, indicating excellent preservation of image fidelity. Furthermore, SNR measurements corroborate the effectiveness of the technique in maintaining signal clarity amidst compression. These results signify a substantial improvement over existing compression techniques, both in terms of file size reduction and retention of image fidelity.

The entropy analysis across various parameters showed that the information content of the reconstructed slices was closely aligned with the original slices, indicating a successful recovery of data with minimal loss of complexity. The slight reduction in Entropy in the stego slices compared to the original slices was negligible, emphasizing the effectiveness of the method in maintaining image integrity. Moreover, the study's analysis of the overall complexity of the proposed downsampling and upsampling approach showed that while the method involves computational and memory complexities, it effectively balances compression and reconstruction quality. Future work could focus on optimizing the algorithm further, exploring its application in different types of medical imaging data, and extending its utility in various telemedicine scenarios.

## Data Availability

The datasets generated and/or analyzed during the current study are available from the corresponding authors upon request.

## References

[1] Anwar SM, Majid M, Qayyum A, Awais M, Alnowami M, Khan MK (2018). Medical image analysis using convolutional neural networks: A review. J. Med. Syst..

[2] Suetens P (2017). Fundamentals of Medical Imaging.

[3] Park C, You SC, Jeon H, Jeong CW, Choi JW, Park RW (2022). Development and validation of the radiology common data model (R-CDM) for the international standardization of medical imaging data. Yonsei Med. J..

[4] Wake, N., Vincent, J. & Robb, F. Medical imaging technologies and imaging considerations for 3D printed anatomic models, in *3D Printing for the Radiologist*, 11–29 (Elsevier, 2022).

[5] Kissi J, Dai B, Dogbe CS, Banahene J, Ernest O (2020). Predictive factors of physicians’ satisfaction with telemedicine services acceptance. Health Inform. J..

[6] May C, Harrison R, Finch T, MacFarlane A, Mair F, Wallace P (2003). Understanding the normalization of telemedicine services through qualitative evaluation. J. Am. Med. Inform. Assoc..

[7] Sriramakrishnan, P., Kalaiselvi, T., Padmapriya, S., Shanthi, N., Ramkumar, S. & Kalaichelvi, N. An medical image file formats and digital image conversion. *Int. J. Eng. Adv. Technol.* **9**(1S4), 74–78.

[8] Dinu, A., Ganesan, R., Kebede, A. A. & Veerasamy, B. Performance analysis and comparison of medical image compression techniques, in *2016 International Conference on Control, Instrumentation, Communication and Computational Technologies (ICCICCT)*, 738–745 (IEEE, 2016).

[9] Gonzalez-Urquijo, M., Macias-Rodriguez, Y. & Davila-Rivas, J. A. The role of telemedicine and globalization in medical education, in *Advancing Health Education With Telemedicine*, 288–295 (IGI Global, 2022).

[10] Vincent RD (2016). MINC 2.0: A flexible format for multi-modal images. Front. Neuroinform..

[11] Venugopal D, Mohan S, Raja S (2016). An efficient block based lossless compression of medical images. Optik.

[12] Rahman MA, Hamada M (2023). A prediction-based lossless image compression procedure using dimension reduction and Huffman coding. Multimed. Tools Appl..

[13] Boopathiraja S, Punitha V, Kalavathi P, Prasath VS (2022). Computational 2D and 3D medical image data compression models. Arch. Comput. Methods Eng..

[14] Rajna, Z., Keskinarkaus, A., Kiviniemi, V. & Seppänen, T. Speeding up the file access of large compressed nifti neuroimaging data, in *37th Annual International Conference of the IEEE Engineering in Medicine and Biology Society (EMBC)*, 654–657 (IEEE, 2015). 10.1109/EMBC.2015.7318447 26736347

[15] Punitha V, Kalavathi P (2020). Analysis of file formats and lossless compression techniques for medical images. Int. J. Sci. Res. Comput..

[16] Naga Srinivasu P, Krishna TB, Ahmed S, Almusallam N, Khaled Alarfaj F, Allheeib N (2023). Variational autoencoders-basedself-learning model for tumor identification and impact analysis from 2-D MRI images. J. Healthc. Eng..

[17] Larobina M, Murino L (2014). Medical image file formats. J. Digit. Imaging.

[18] Clarke WT (2022). NIfTI-MRS: A standard data format for magnetic resonance spectroscopy. Magn. Reson. Med..

[19] Kim D-W, Choi J-Y, Han K-H, Making D (2020). Risk management-based security evaluation model for telemedicine systems. BMC Med. Inform..

[20] Zuev, M. & Enyagina, I. System for storing and analyzing experimental MRI/fMRI data on the hybrilit heterogeneous platform, in *Proceedings of the Information System for the Tasks of Radiation Biology Workshop* (2020).

[21] Larobina M, Murino L (2014). Medical image file formats. J. Digit. Imaging.

[22] Fink A (2021). A two-week running intervention reduces symptoms related to depression and increases hippocampal volume in young adults. Cortex.

[23] Andreas Fink KK, Zussner T, Perchtold-Stefan CM, Rominger C, Benedek M, Papousek I (2021). A Two-Week Running Intervention Reduces Symptoms Related to Depression and Increases Hippocampal Volume in Young Adults. Cortex.

[24] Keune PM, Bostanov V, Kotchoubey B, Hautzinger M (2012). Mindfulness versus rumination and behavioral inhibition: A perspective from research on frontal brain asymmetry. Personal. Individ. Differ..

[25] Whitcher B, Schmid VJ, Thorton A (2011). Working with the DICOM and NIfTI Data Standards in R. J. Stat. Softw..

[26] Li X, Morgan PS, Ashburner J, Smith J, Rorden C (2016). The first step for neuroimaging data analysis: DICOM to NIfTI conversion. J. Neurosci. Methods.

[27] Clarke, W. T. *et al.* NIfTI-MRS: A standard format for magnetic resonance spectroscopic data. *bioRxiv,* (2021).

[28] Huynh-Thu Q, Ghanbari M (2008). Scope of validity of PSNR in image/video quality assessment. Electron. Lett..

[29] Wang Z, Bovik AC, Sheikh HR, Simoncelli EP (2004). Image quality assessment: From error visibility to structural similarity. IEEE Trans. Image Process..

[30] Elhadad A, Ghareeb A, Abbas S (2021). A blind and high-capacity data hiding of DICOM medical images based on fuzzification concepts. Alex. Eng. J..

[31] Wang Z, Bovik AC (2002). A universal image quality index. IEEE Signal Process. Lett..

[32] Gonzalez RC, Woods RE (2008). Digital image processing: Pearson prentice hall. Upper Saddle River NJ.

[33] Gonzalez, R., Woods, R. & Eddins, S. 11 Representation and description, in *Digital Image Processing Using MATLAB* (Prentice-Hall Englewood Cliffs, 2003)

[34] Williamson DF, Parker RA, Kendrick JS (1989). The box plot: A simple visual method to interpret data. Ann. Intern. Med..

[35] Potter K, Hagen H, Kerren A, Dannenmann P (2006). Methods for presenting statistical information: The box plot. Vis. Large Unstruct. Data Sets.

[36] Anelli, V. W., Deldjoo, Y., Di Noia, T. & Malitesta, D. Deep learning-based adaptive image compression system for a real-world scenario, in *2020 IEEE Conference on Evolving and Adaptive Intelligent Systems (EAIS)* 1–8 (IEEE, 2020).

[37] Li Z (2023). Nearly-lossless-to-lossy medical image compression by the optimized JPEGXT and JPEG algorithms through the anatomical regions of interest. Biomed. Signal Process. Control.

